# Supplementary material for: Assessing the impact of artifact correction and artifact rejection on the performance of SVM- and LDA-based decoding of EEG signals
Source: Neuroimage. Author manuscript; Available in PMC 2026 Jun 3. (PMC13232936; doi:10.1016/j.neuroimage.2025.121304)
Supplement: 1 [file NIHMS2177245-supplement-1.pdf]

Supplementary Materials for Assessing the impact of artifact correction and artifact rejection on the performance of SVM-based decoding of EEG signals

Guanghui Zhang[a,b,c,*], Steven J. Luck[c]

[a] *Research Center of Brain and Cognitive Neuroscience, Liaoning Normal University, Dalian, Liaoning, China*
[b] *Key Laboratory of Brain and Cognitive Neuroscience, Liaoning Province, Dalian, China*
[c] *Center for Mind & Brain, University of California-Davis, Davis, CA, USA*

Contents

1 Effect of low-pass filter on the decoding performance 2

2 Random trial removal to assess impact of artifact rejection thresholds 5

3 Regularized LDA decoding 8

4 Effect of normalization on decoding performance 11

5 Quantifying Noise 14

*Corresponding author
Email addresses: zhang.guanghui@foxmail.com (Guanghui Zhang), sjluck@ucdavis.edu (Steven J. Luck)

1. Effect of low-pass filter on the decoding performance

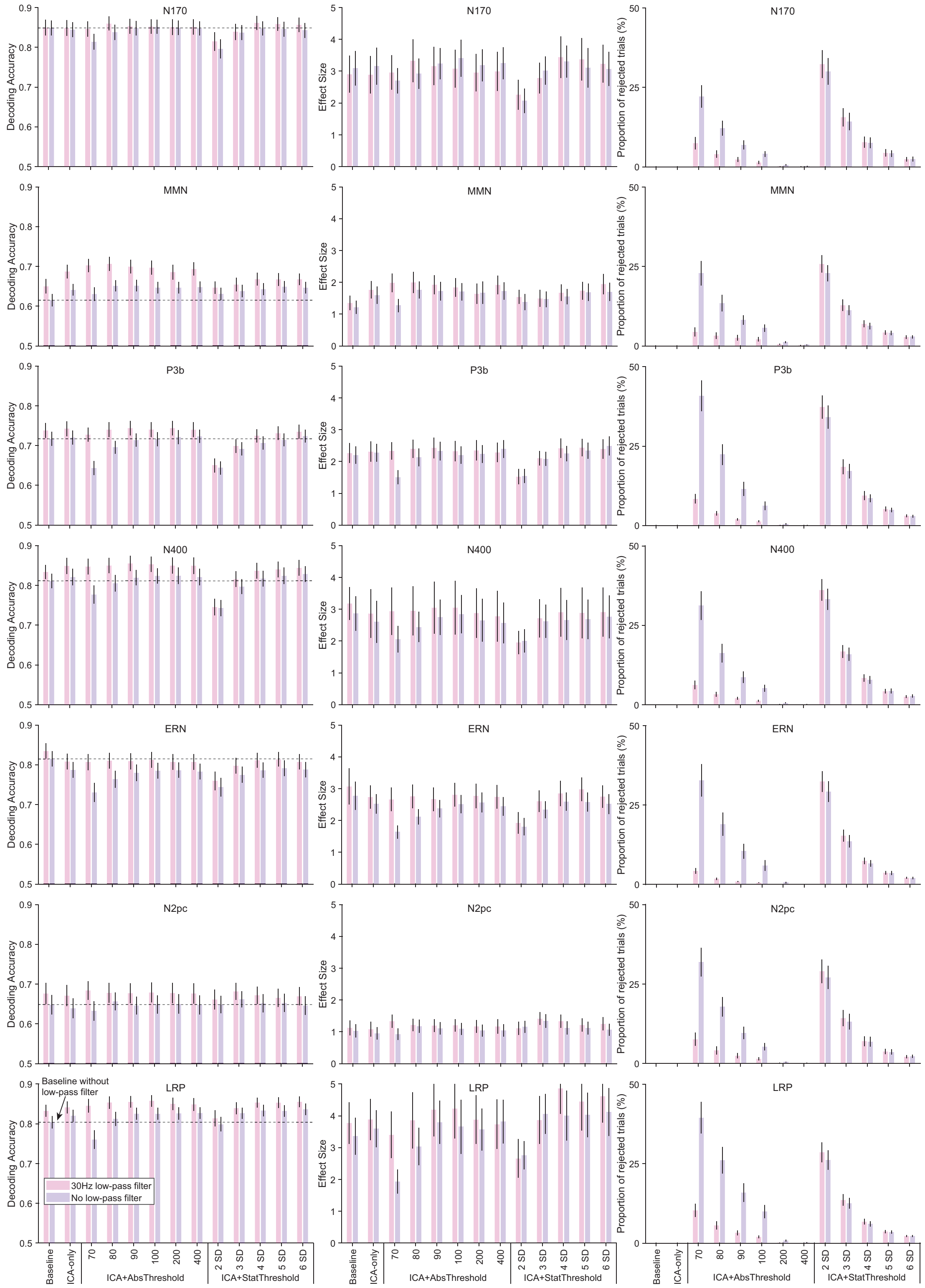

Figure S1: Decoding accuracy (left column), effect size (Cohen's dz, middle column), and percentage of rejected trials (right column) for the data filtered with or without a 30 Hz low pass filter for the ERP CORE experiments.

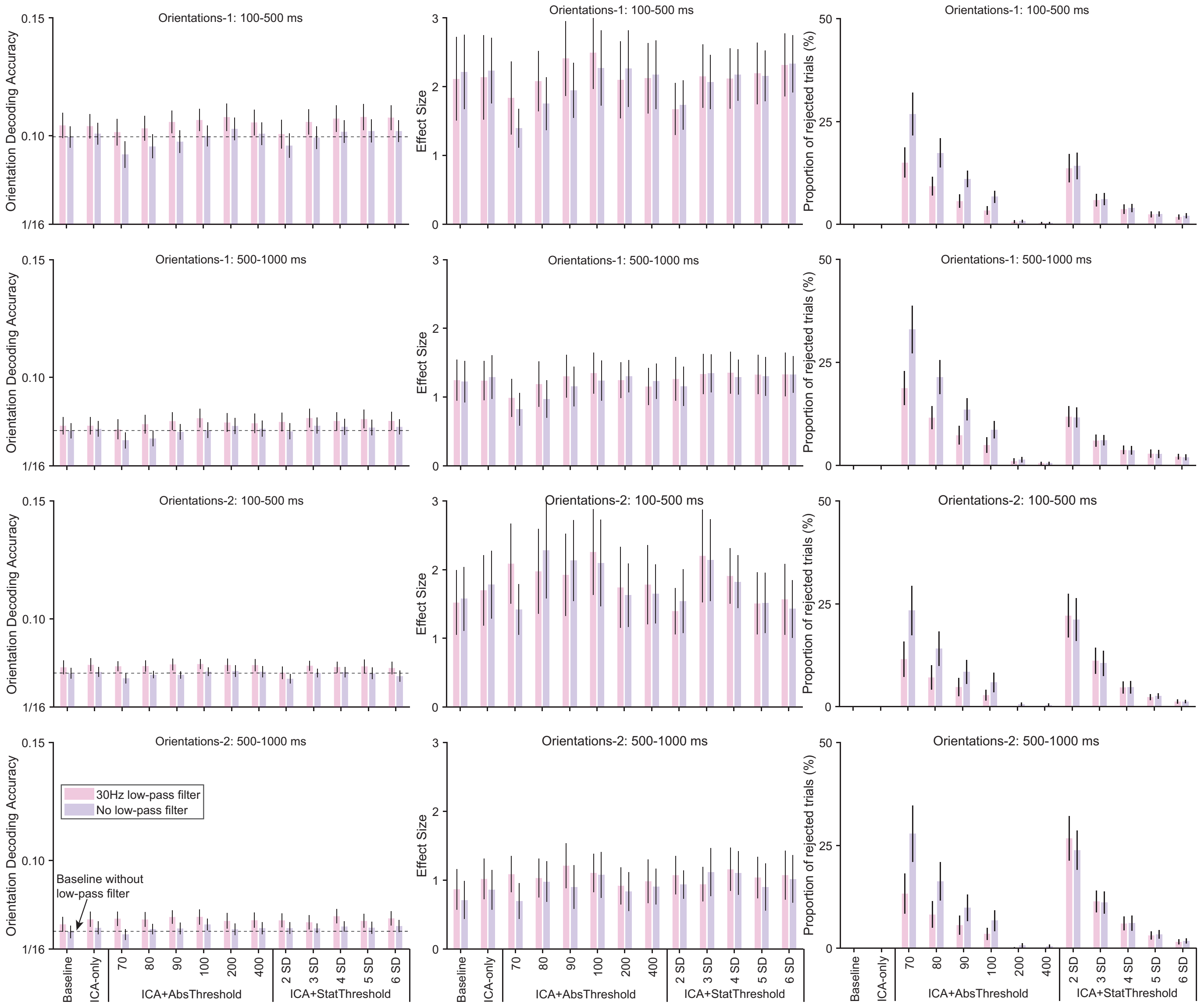

Figure S2: Decoding accuracy (left column), effect size (Cohen's dz, middle column), and percentage of rejected trials (right column) resulting from the different artifact minimization approaches for the Orientations-1 and Orientations-2 datasets with or without 30Hz low-pass filter , separately for a perception time-window (100-500 ms) and a working memory maintenance time window (500-1000 ms).

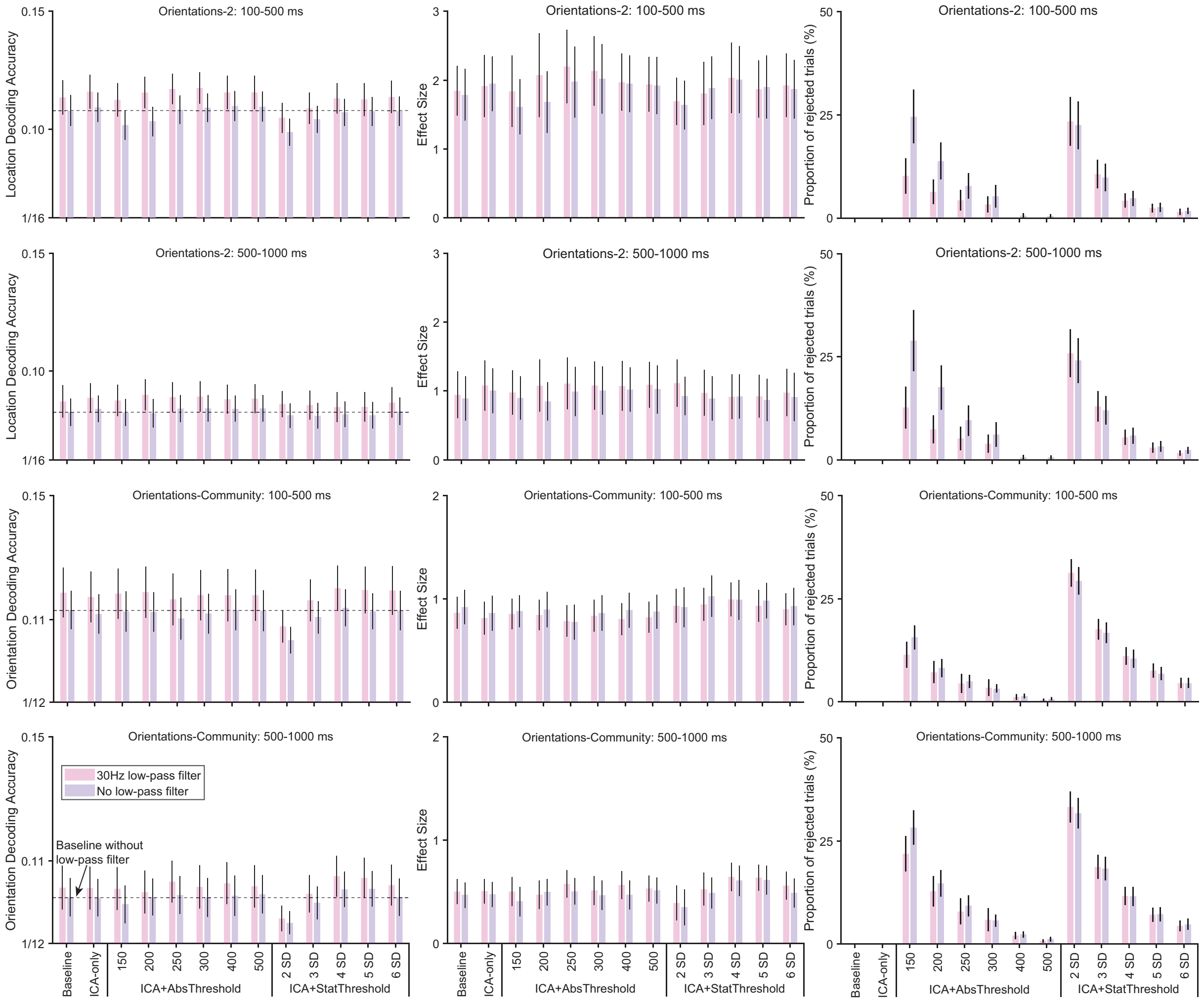

Figure S3: Decoding accuracy (left column), effect size (Cohen's dz, middle column), and percentage of rejected trials (right column) resulting from the different artifact minimization approaches for decoding of stimulus location in the Orientations-2 dataset and decoding of orientation in the Orientations-Community dataset with or without 30Hz low-pass filter, separately for a perception time-window (100-500 ms) and a working memory maintenance time window (500-1000 ms).

2. Random trial removal to assess impact of artifact rejection thresholds

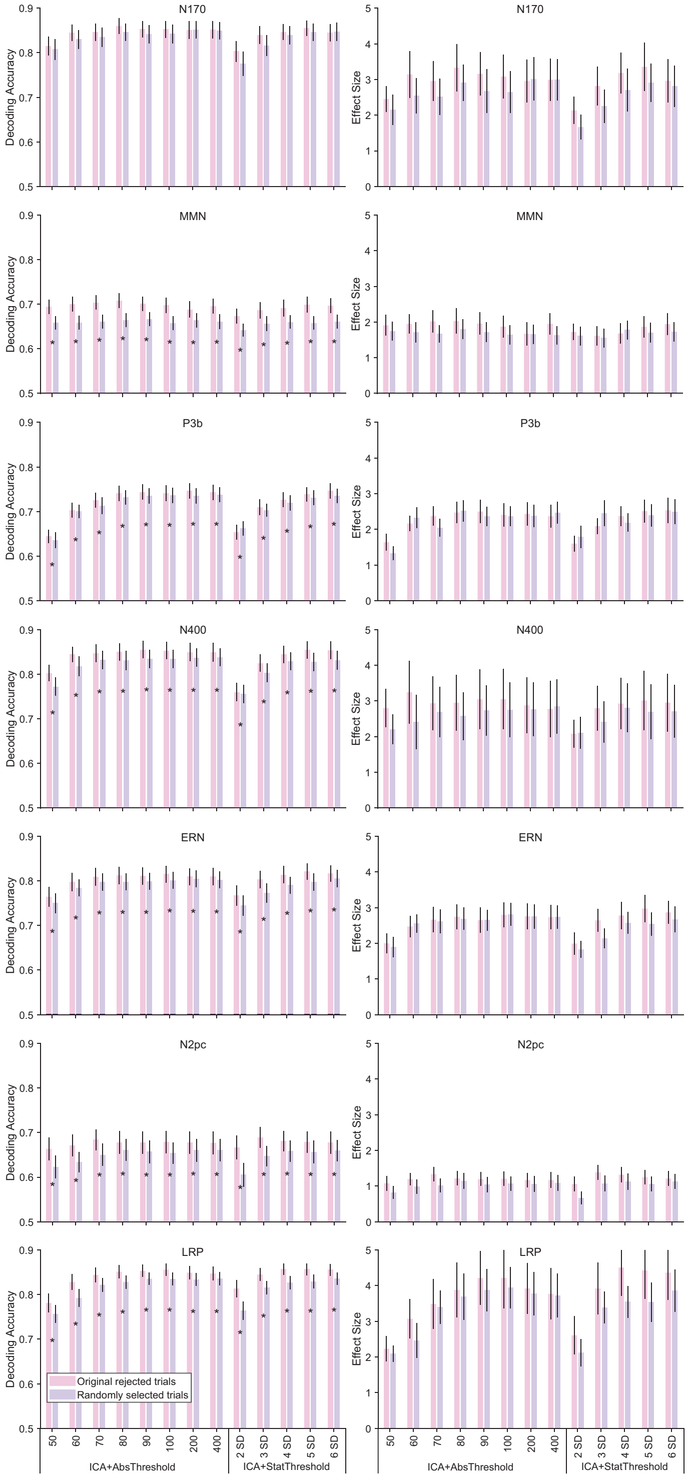

Figure S4: Decoding accuracy (left column) and effect size (Cohen's dz, right column) for the data for ERP CORE experiments for trial removal using either an artifact rejection algorithm or random selection.

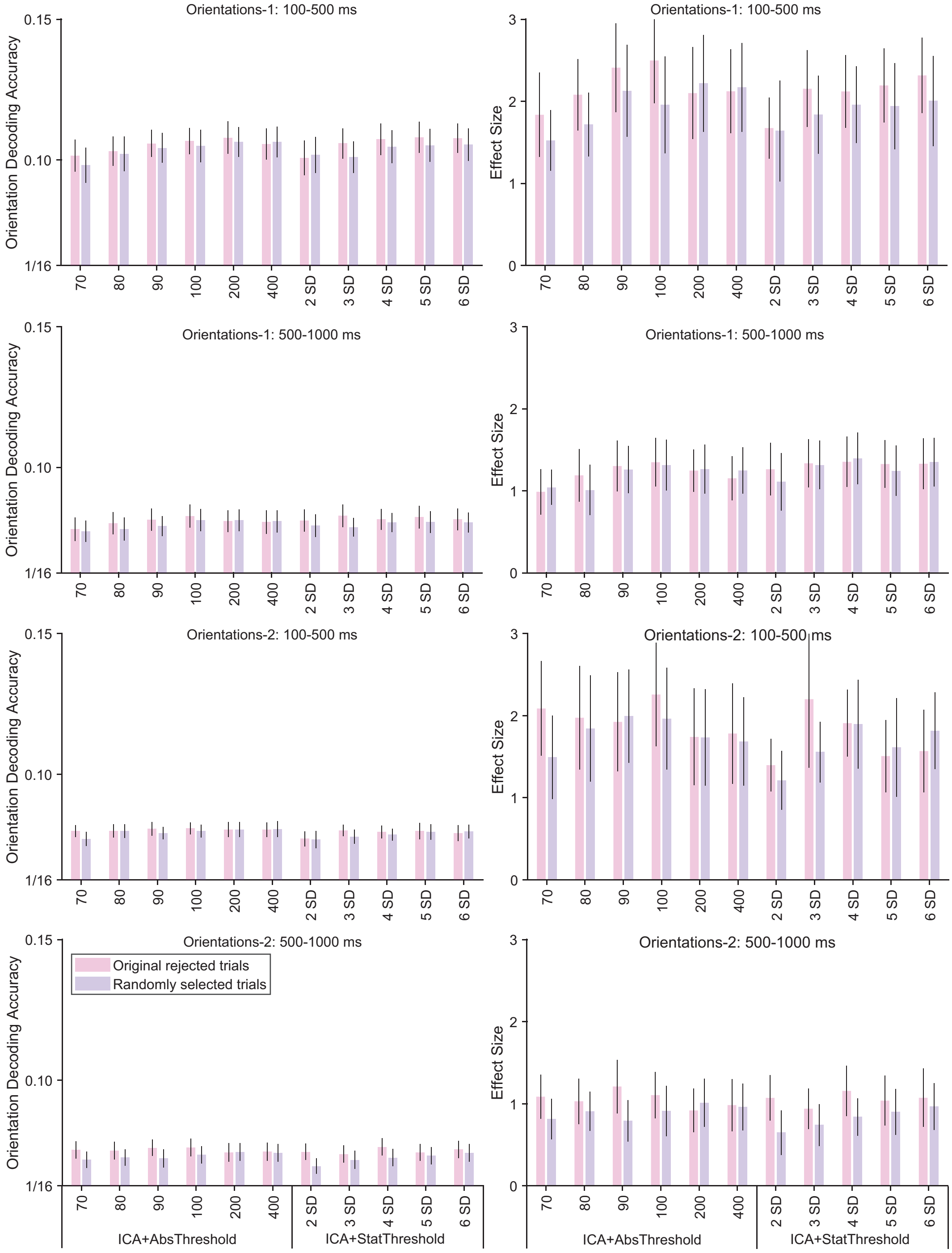

Figure S5: Decoding accuracy (left column) and effect size (Cohen's dz, right column) resulting from the different artifact minimization approaches for the Orientations-1 and Orientations-2 datasets, separately for a perception time-window (100-500 ms) and a working memory maintenance time window (500-1000 ms) when trials were removed using either an artifact rejection algorithm or random selection.

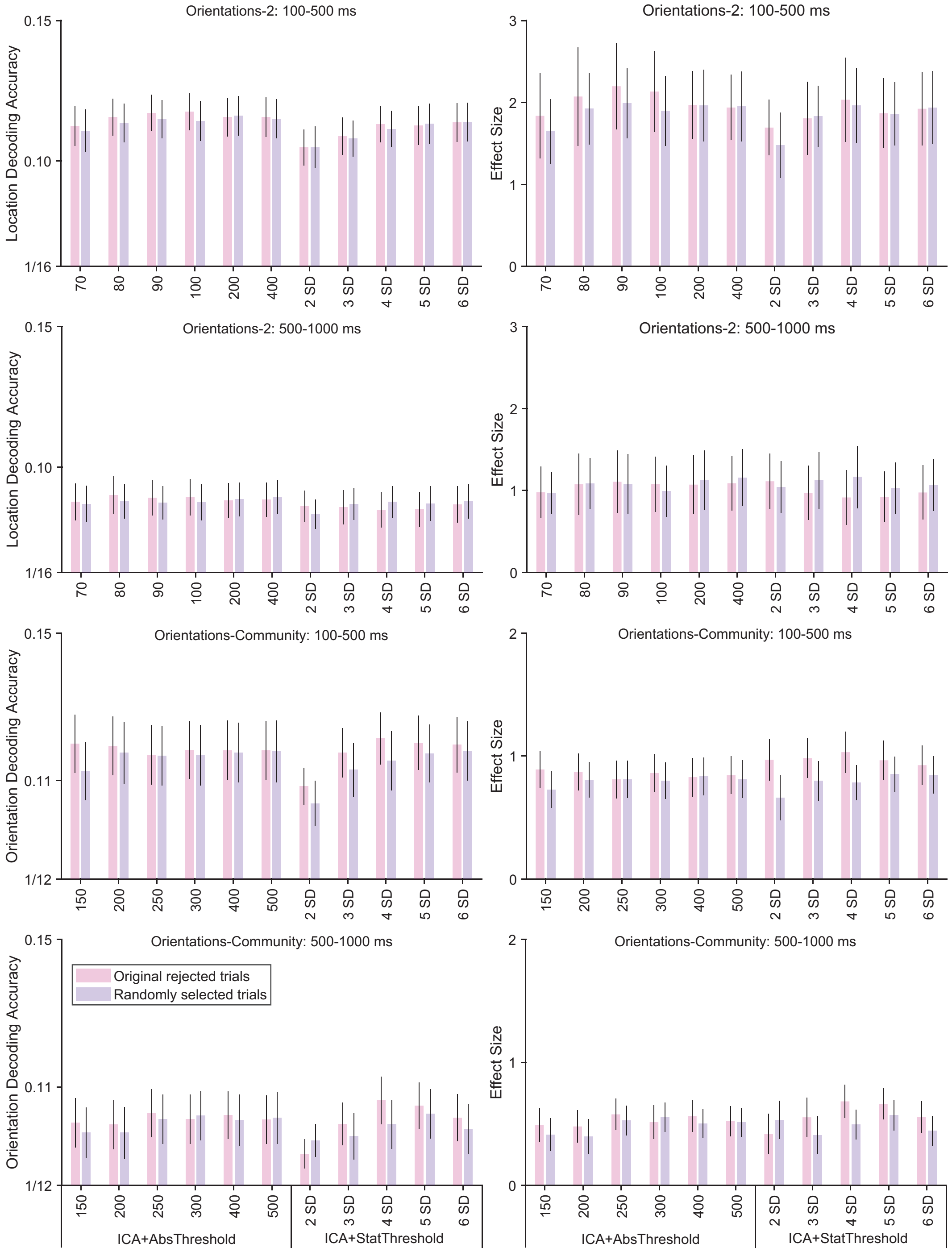

Figure S6: Decoding accuracy (left column) and effect size (Cohen's dz, right column) resulting from the different artifact minimization approaches for decoding of stimulus location in the Orientations-2 dataset and decoding of orientation in the Orientations-Community dataset, separately for a perception time-window (100-500 ms) and a working memory maintenance time window (500-1000 ms) when trials were removed using either an artifact rejection algorithm or random selection.

3. Regularized LDA decoding

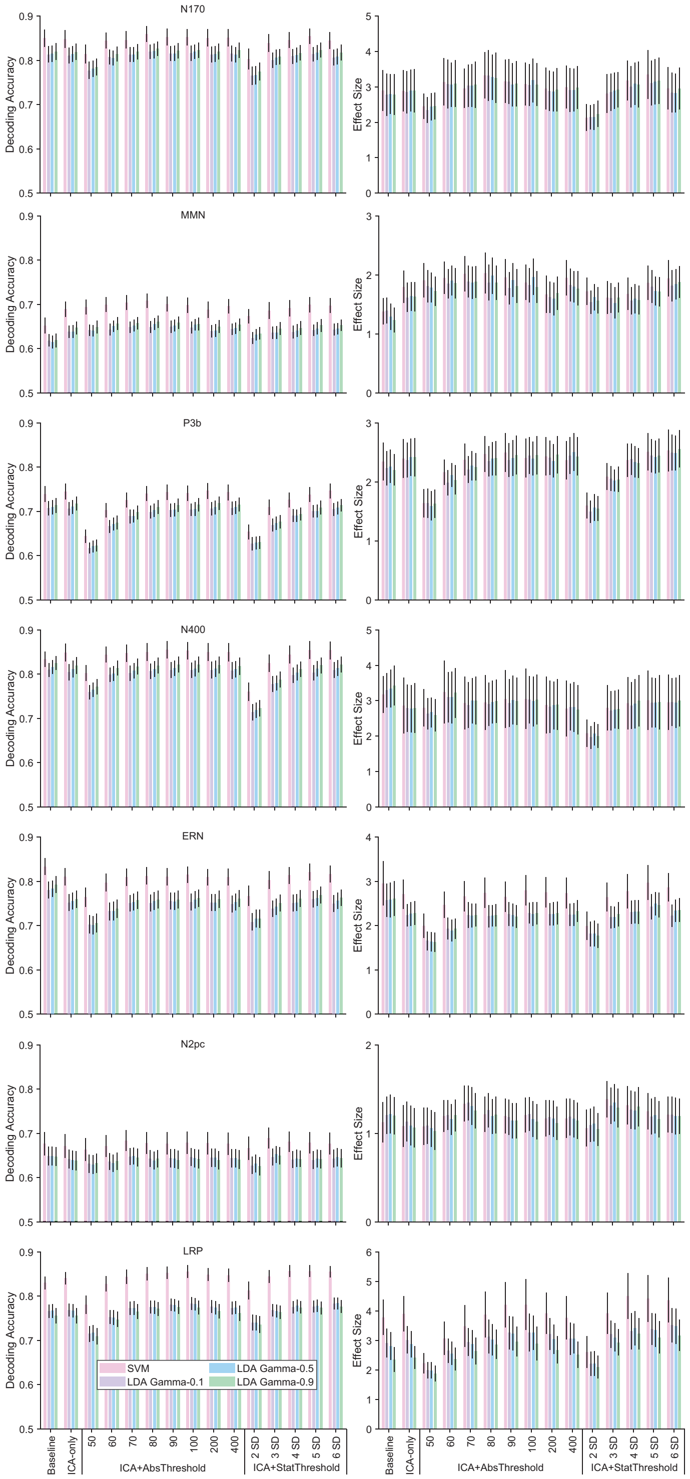

Figure S7: Decoding accuracy (left column) and effect size (Cohen's dz, right column) for the ERP CORE data, comparing SVM with regularized LDA using different regularization parameters (Gamma = 0.1, 0.5, and 0.9).

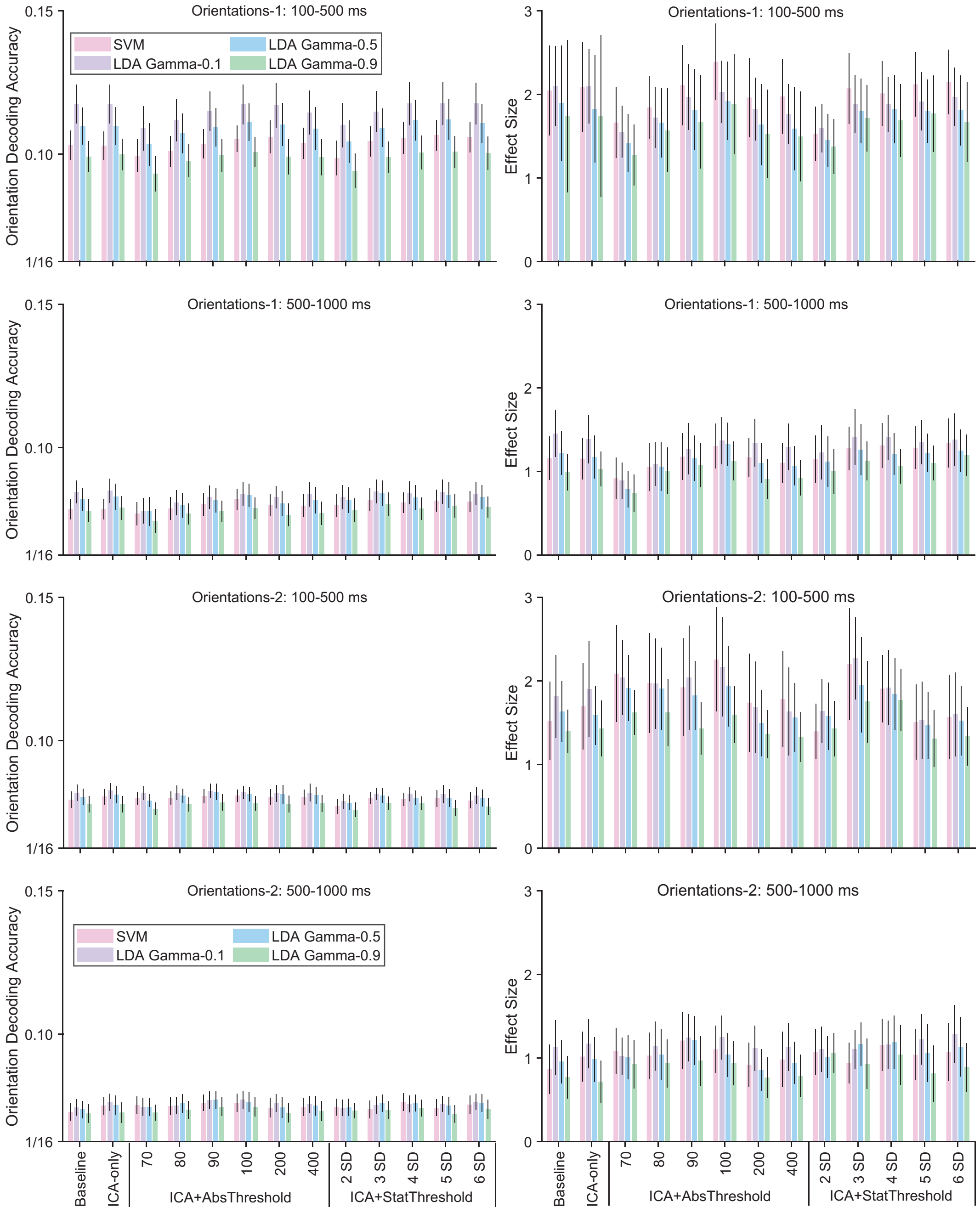

Figure S8: Decoding accuracy (left column) and effect size (Cohen's dz, right column) resulting from the different artifact minimization approaches for the Orientations-1 and Orientations-2 datasets, comparing SVM with regularized LDA using different regularization parameters (gamma = 0.1,0.5, and 0.9), separately for a perception time-window (100-500 ms) and a working memory maintenance time window (500-1000 ms).

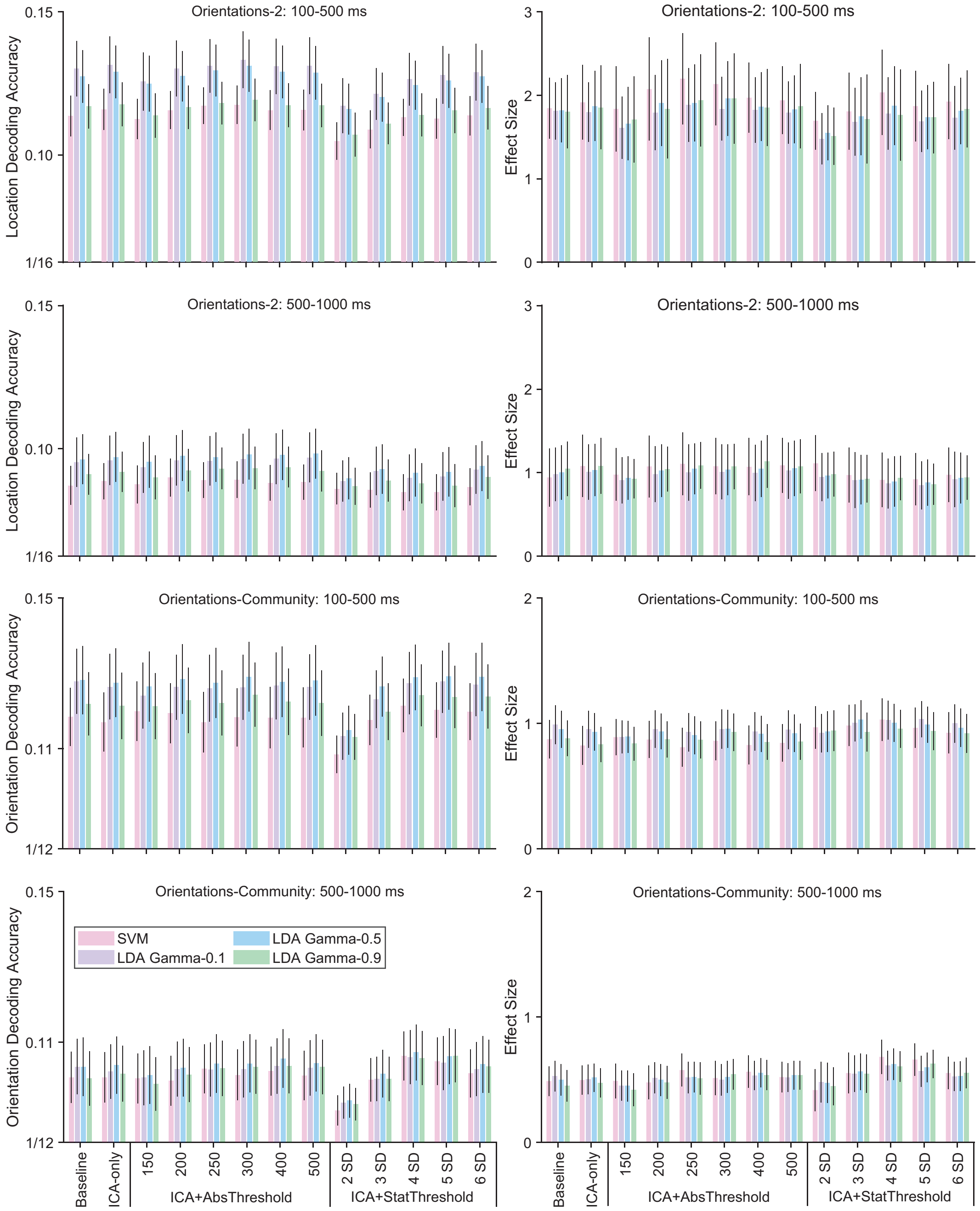

Figure S9: Decoding accuracy (left column) and effect size (Cohen's dz, right column) resulting from the different artifact minimization approaches for decoding of stimulus location in the Orientations-2 dataset and decoding of orientation in the Orientations-Community dataset, comparing SVM with regularized LDA using different regularization parameters (gamma = 0.1, 0.5, and 0.9), separately for a perception time-window (100-500 ms) and a working memory maintenance time window (500-1000 ms).

4. Effect of normalization on decoding performance

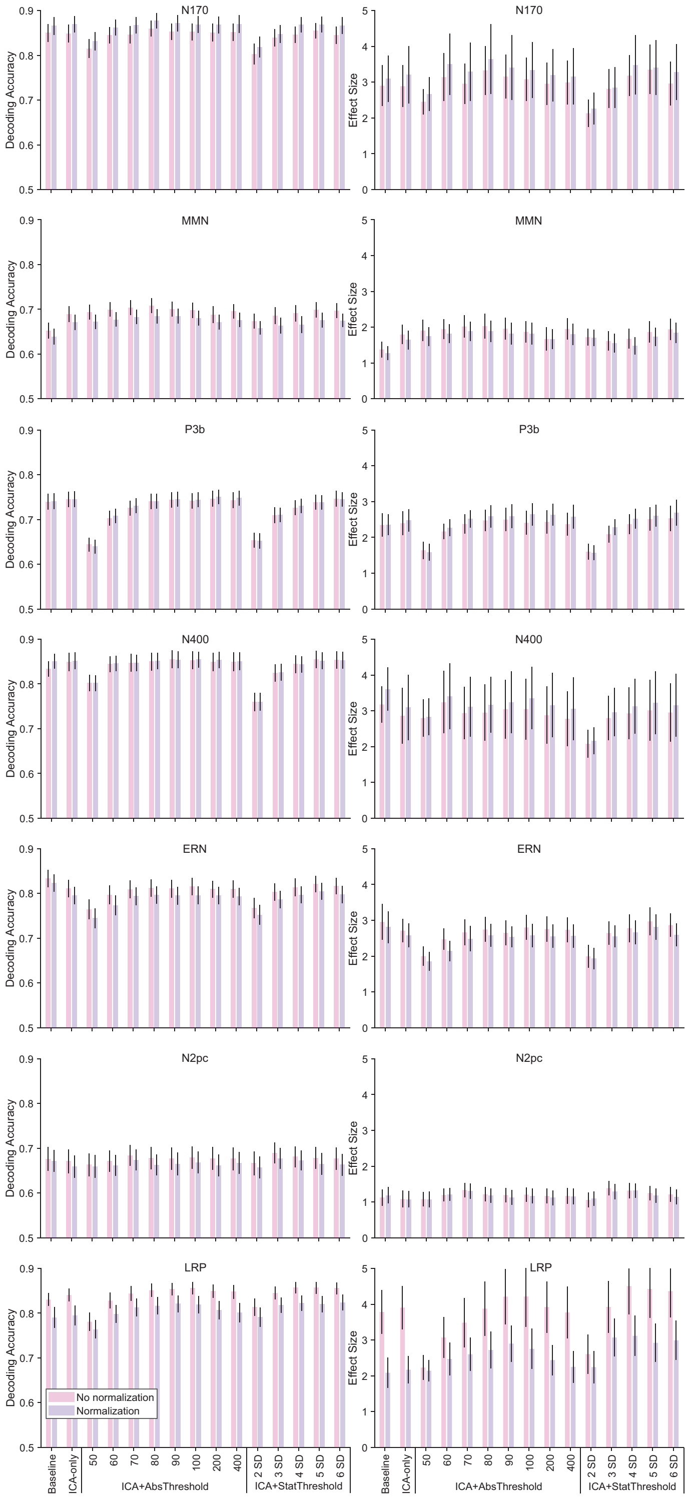

Figure S10: Decoding accuracy (left column) and effect size (Cohen's dz, right column) for the data with or without normalization for the ERP CORE experiments.

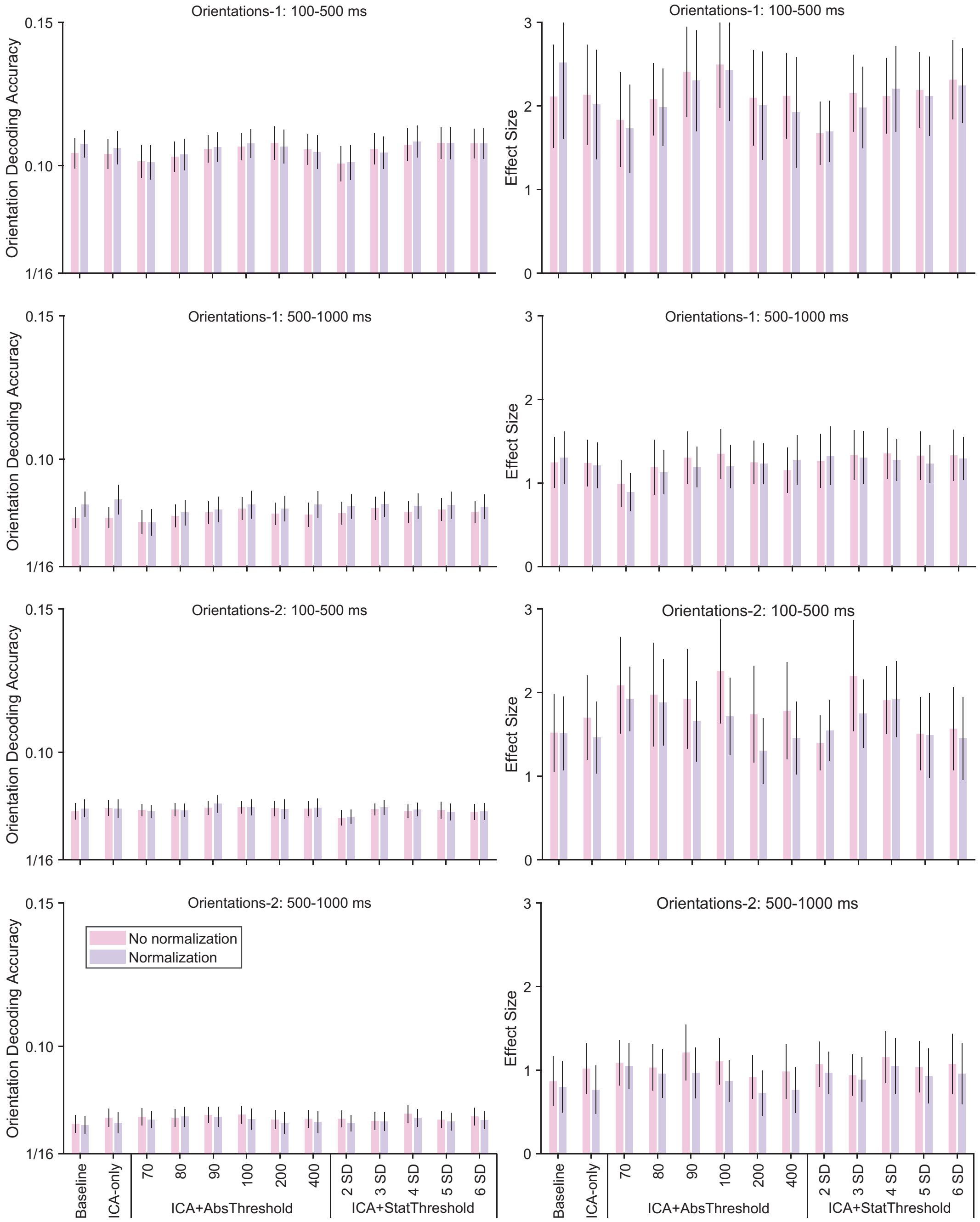

Figure S11: Decoding accuracy (left column), effect size (Cohen's dz, middle column), and percentage of rejected trials (right column) resulting from the different artifact minimization approaches for the Orientations-1 and Orientations-2 datasets with or without normalization, separately for a perception time-window (100-500 ms) and a working memory maintenance time window (500-1000 ms).

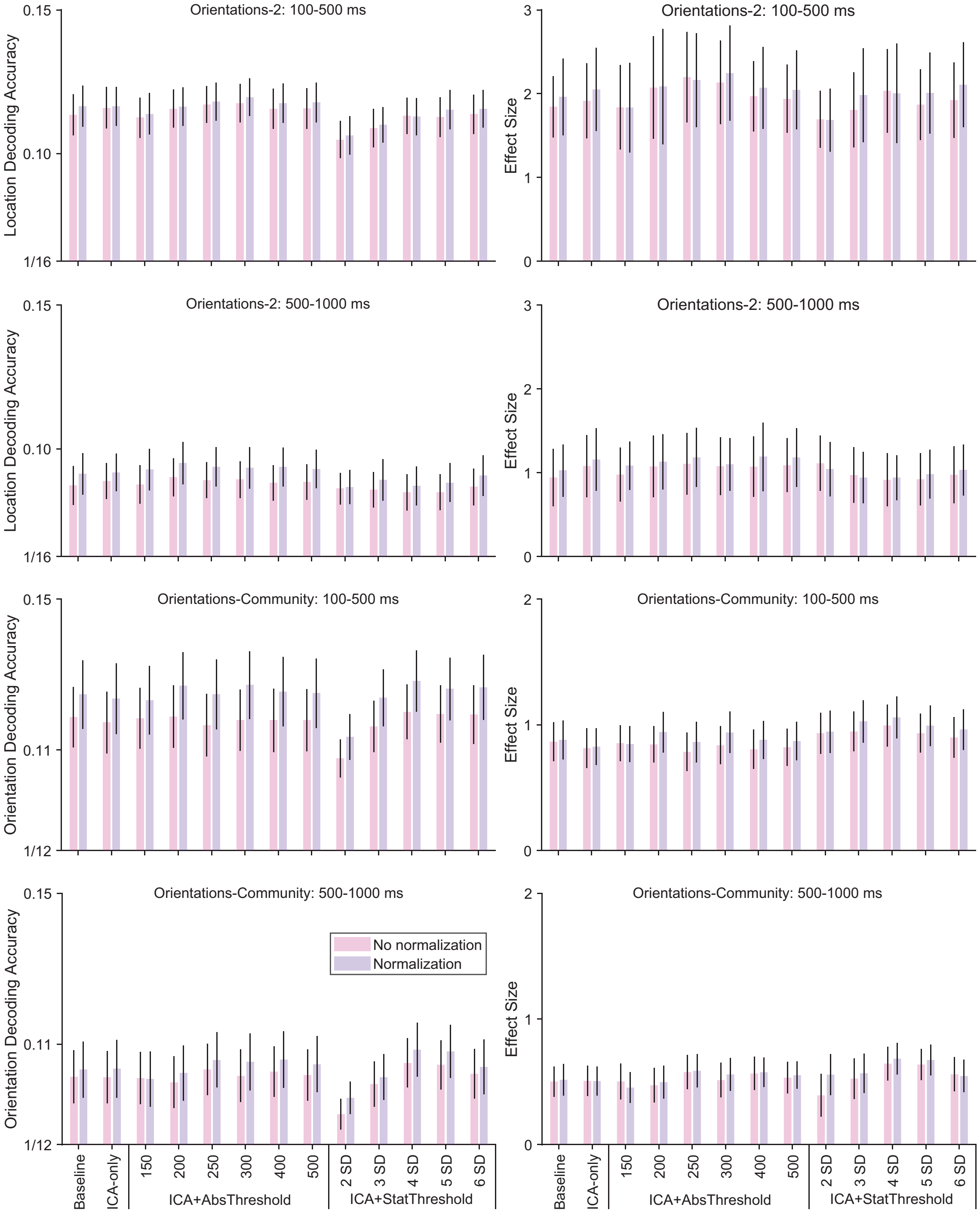

Figure S12: Decoding accuracy (left column), effect size (Cohen's dz, middle column), and percentage of rejected trials (right column) resulting from the different artifact minimization approaches for decoding of stimulus location in the Orientations-2 dataset and decoding of orientation in the Orientations-Community dataset with or without normalization, separately for a perception time-window (100-500 ms) and a working memory maintenance time window (500-1000 ms).

5. Quantifying Noise

Table S1: Mean and 95% confidence interval of the measurement error values for different experiments.

Experiment	Mean measurement error	95% confidence interval	Experiment	Mean measurement error	95% confidence interval
N170	0.83	[0.77, 0.90]	Orientations-1, 100-500	1.67	[1.25, 2.09]
MMN	0.82	[0.55, 1.09]	Orientations-1, 500-1000	2.21	[1.39, 3.04]
P3b	1.58	[1.42, 1.73]	Orientations-2, (orientation decoding), 100-500	1.38	[1.21, 1.54]
N400	1.58	[1.46, 1.70]	Orientations-2, (orientation decoding), 500-1000	1.59	[1.43, 1.75]
ERN	1.40	[1.24, 1.57]	Orientations-2, (location decoding) 100-500	1.36	[1.20, 1.53]
N2pc	0.90	[0.82, 0.98]	Orientations-2, (location decoding) 500-1000	1.59	[1.43, 1.75]
LRP	0.98	[0.88, 1.08]	Orientations-Community, 100-500	2.20	[1.43, 2.97]
			Orientations-Community, 500-1000	2.80	[1.52, 4.08]

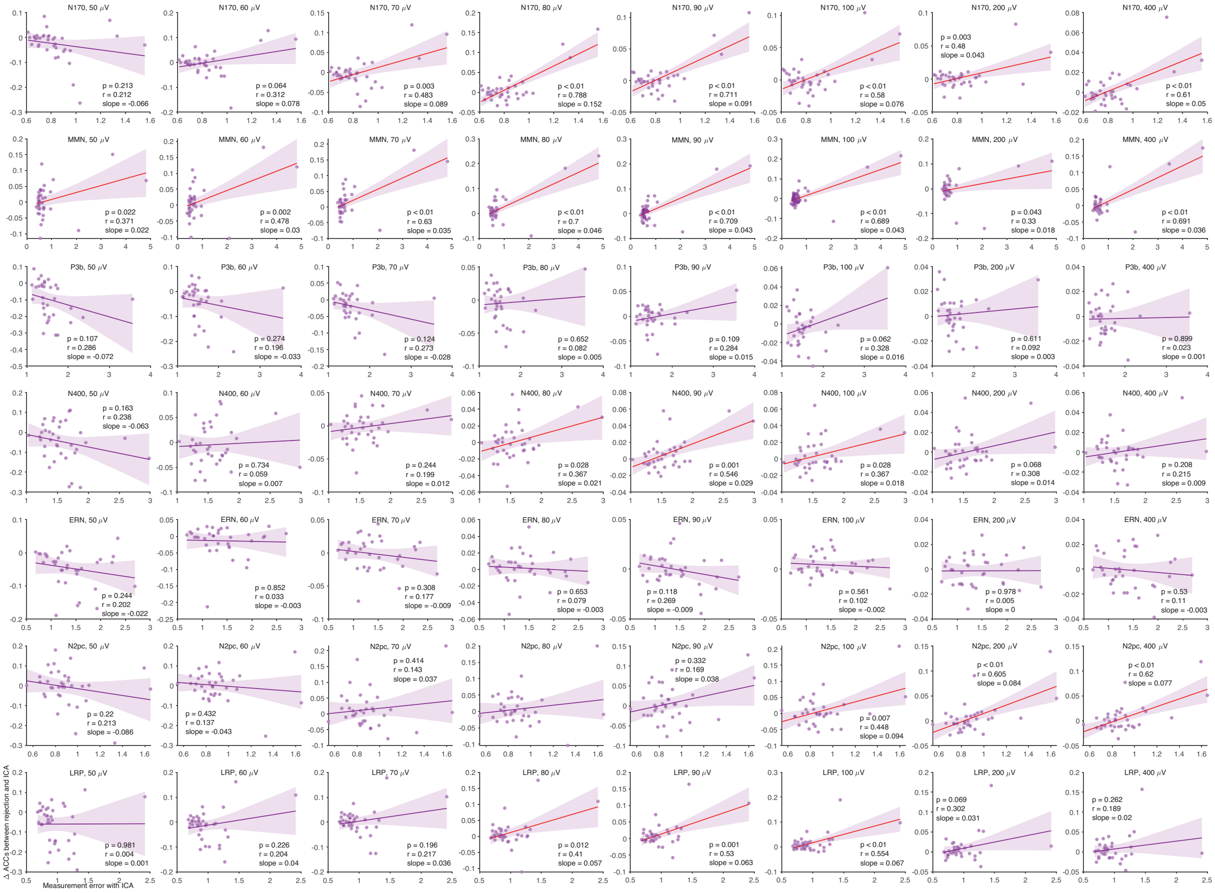

Figure S13: Scatterplots of the relationship between measurement error (X axis) and the difference in decoding accuracy between the ICA+AbsThreshold approach and the ICA-Only approach (Y axis) for different rejection thresholds and each of the ERP components from the ERP CORE. Note that we used the simple voltage threshold (SVT) algorithm and moving window peak-to-peak (MWP) algorithm in ERPLAB to detect and reject trials with artifacts with thresholds of 50, 60, 70, 80, 90, 100, 200, or 400 µV. To estimate the measurement error, we first computed the standard error of the mean (SEM) of the amplitudes across trials at each time point within the measured time window (see Table 1 for time window for each component) for each experimental condition in a given participant, and we then calculated the root mean square of these SEM values.

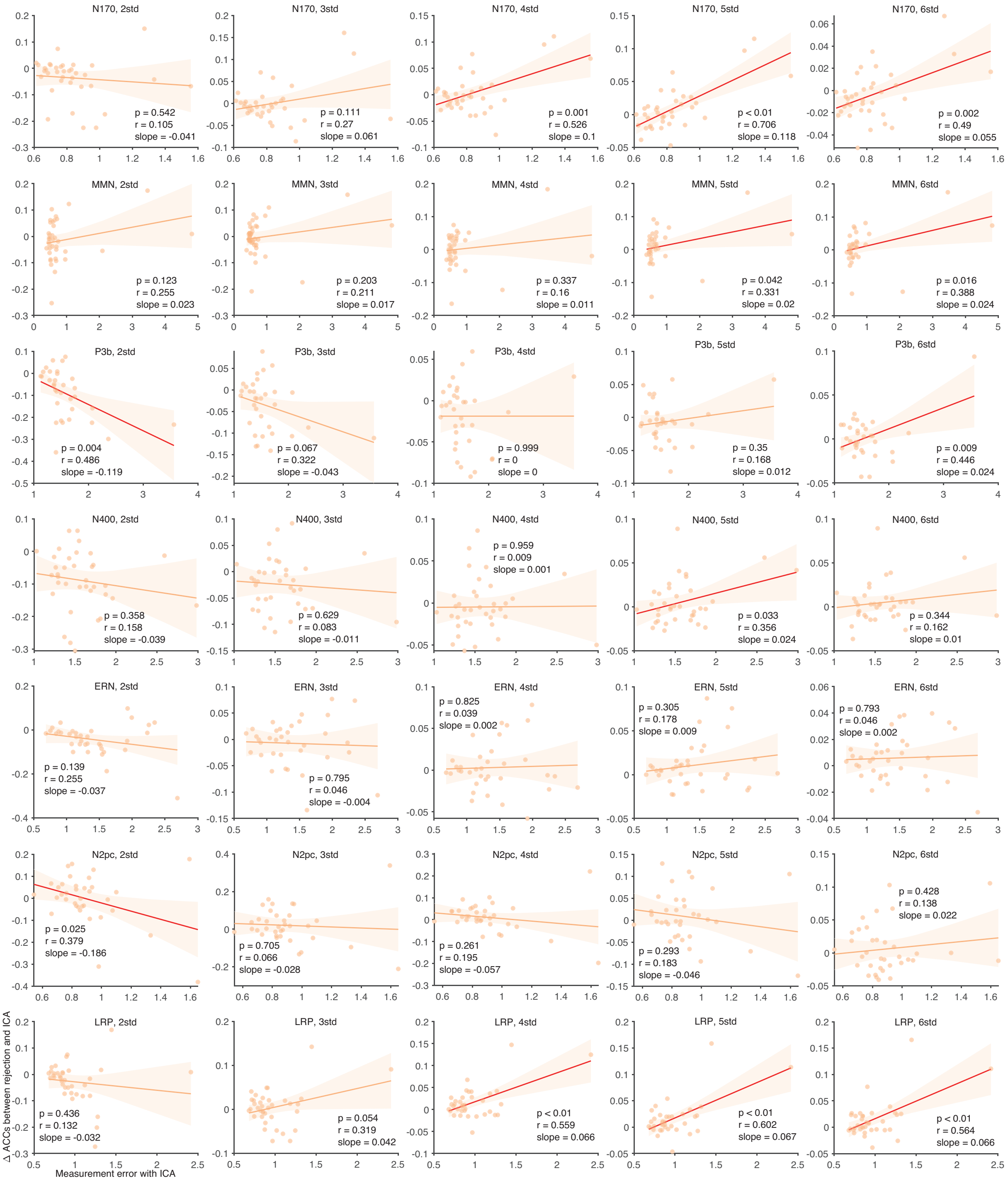

Figure S14: Scatterplots of the relationship between measurement error (X axis) and the difference in decoding accuracy between the ICA+StatThreshold approach and the ICA-Only approach (Y axis) for different rejection thresholds and each of the ERP components from ERP CORE. Note that we used the probability density algorithm to detect and reject trials with artifacts with thresholds of 2 ,3, 4, 5, or 6 standard deviations (SDs) from the mean.

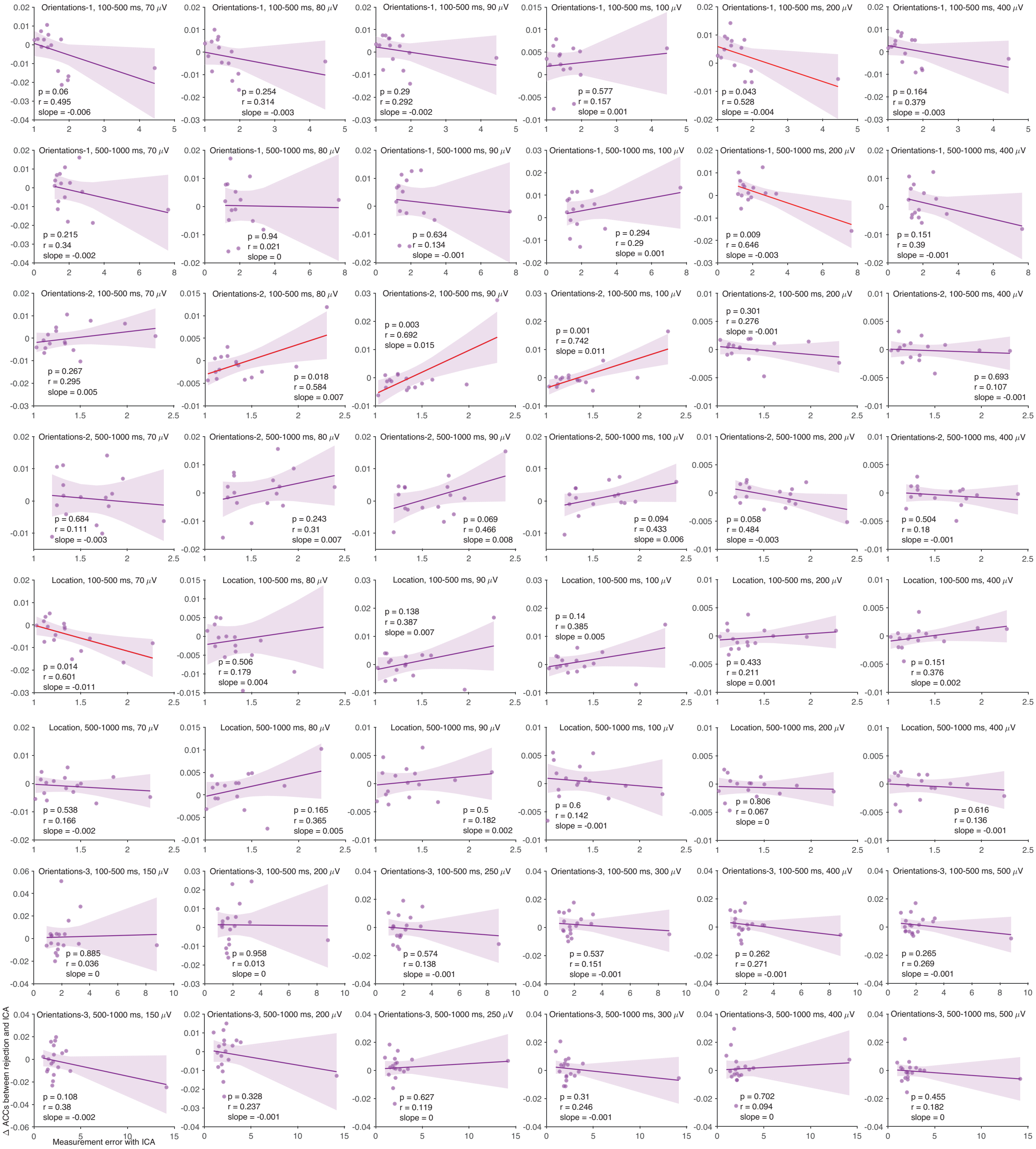

Figure S15: Scatterplots of the relationship between measurement error (X axis) and the difference in decoding accuracy between the ICA+AbsThreshold approach and the ICA-Only approach (Y axis) for different rejection thresholds and each of the ERP components from the Orientations-1, Orientations-2, and Orientations-Community experiments. Note that we used the simple voltage threshold (SVT) algorithm and moving window peak-to-peak (MWP) algorithm in ERPLAB to detect and reject trials with artifacts with thresholds of 50, 60, 70, 80, 90, 100, 200, or 400 µV.

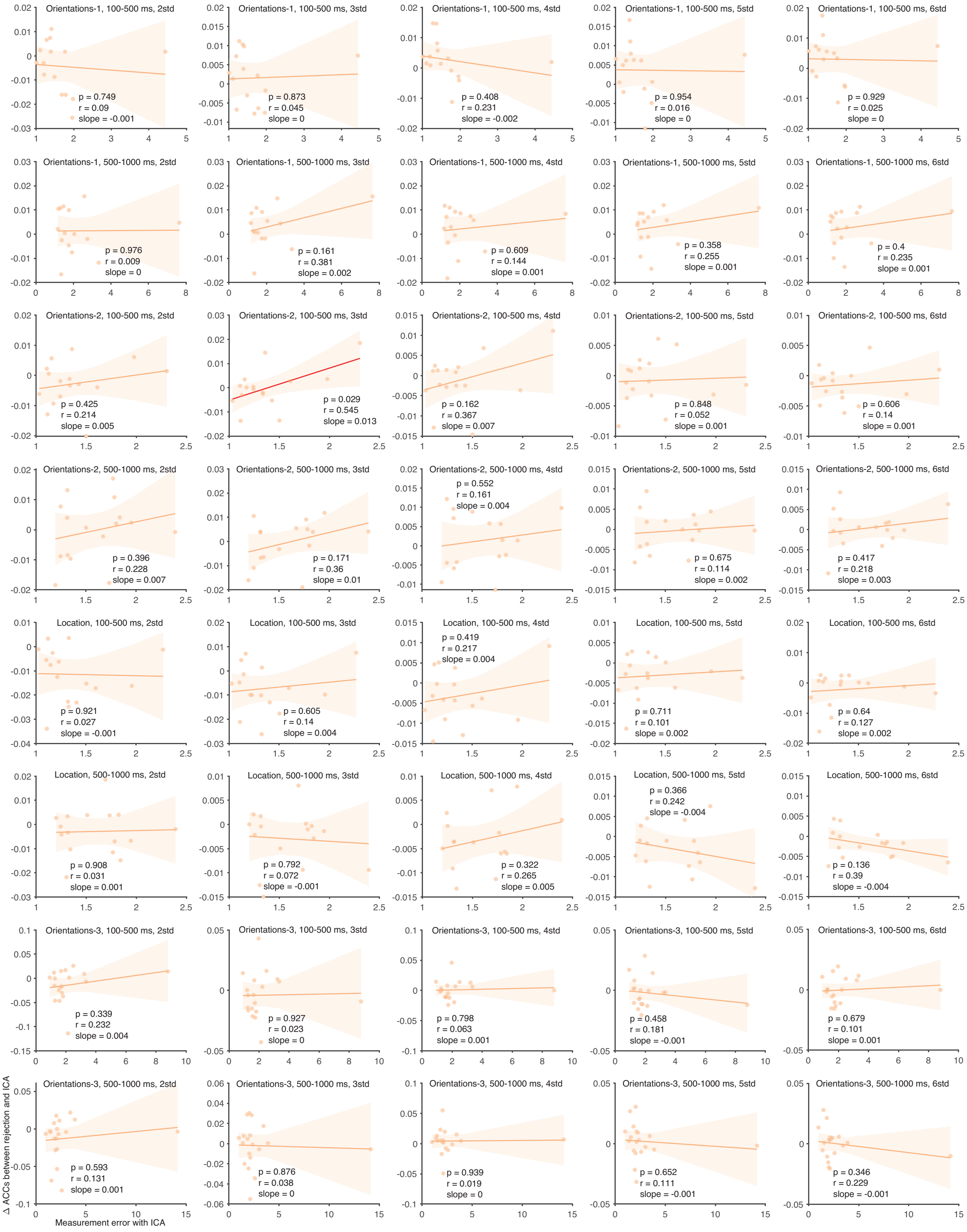

Figure S16: Scatterplots of the relationship between measurement error (X axis) and the difference in decoding accuracy between the ICA+StatThreshold approach and the ICA-Only approach (Y axis) for different rejection thresholds and each of the ERP components from the Orientations-1, Orientations-2, and Orientations-Community experiments. Note that we used the probability density algorithm to detect and reject trials with artifacts with thresholds of 2, 3, 4, 5, or 6 standard deviations (SDs) from the mean.
